# Rhizo-lysimetry: facilities for the simultaneous study of root behaviour and resource use by agricultural crop and pasture systems

**DOI:** 10.1186/1746-4811-9-3

**Published:** 2013-01-31

**Authors:** Philip L Eberbach, Jeffrey Hoffmann, Sergio J Moroni, Leonard J Wade, Leslie A Weston

**Affiliations:** 1 EH Graham Centre for Agricultural Innovation, School of Agricultural and Wine Sciences, Charles Sturt University, Boorooma St, 2678, Wagga Wagga, NSW, Australia; 2 , 2656, Ceranya, Lockhart, NSW, Australia

**Keywords:** Rhizo-lysimeter, Time Domain Reflectometry (TDR), Mini-rhizotron-root observation tube, Root growth, Lucerne, Canola, Wheat

## Abstract

**Background:**

Rhizo-lysimeters offer unique advantages for the study of plants and their interactions with soils. In this paper, an existing facility at Charles Sturt University in Wagga Wagga Australia is described in detail and its potential to conduct both ecophysiological and ecohydrological research in the study of root interactions of agricultural crops and pastures is quantitatively assessed. This is of significance to future crop research efforts in southern Australia, in light of recent significant long-term drought events, as well as potential impacts of climate change as predicted for the region. The rhizo-lysimeter root research facility has recently been expanded to accommodate larger research projects over multiple years and cropping rotations.

**Results:**

Lucerne, a widely-grown perennial pasture in southern Australia, developed an expansive root system to a depth of 0.9 m over a twelve month period. Its deeper roots particularly at 2.05 m continued to expand for the duration of the experiment. In succeeding experiments, canola, a commonly grown annual crop, developed a more extensive (approximately 300%) root system than wheat, but exhibited a slower rate of root elongation at rates of 7.47 x 10^–3^ m day^–1^ for canola and 1.04 x10^–2^ m day^–1^ for wheat. A time domain reflectometry (TDR) network was designed to accurately assess changes in soil water content, and could assess water content change to within 5% of the amount of water applied.

**Conclusions:**

The rhizo-lysimetry system provided robust estimates of root growth and soil water change under conditions representative of a field setting. This is currently one of a very limited number of global research facilities able to perform experimentation under field conditions and is the largest root research experimental laboratory in the southern hemisphere.

## Background

A major challenge facing plant scientists and ecologists is achieving a greater understanding of the behaviour of plant roots, particular when growing in undisturbed field soils, and how of these organs respond to various resources or stimuli. Traditional knowledge of the effect of a particular treatment on plant roots has largely been derived from destructive samplings, yet a more comprehensive understanding of the relationship between plant roots and applied stimuli can be gained from non-destructive, repeated observations of root activity. There is an increasing need to develop improved methods to enable *in situ* observations of plant roots in response to soil treatments

Current methods to quantify root behaviour *in situ* have been reviewed [1,2]. Approaches that do not involve the destructive harvest of plant tissue are rare, but include rhizotron techniques; specifically the use of root windows (flat surfaces) or mini-rhizotrons (curved surfaces) [1,3,4], or root imaging techniques, such as computer aided tomography or magnetic resonance [5]. While imaging techniques have good spatial resolution, they can only scan comparatively small volumes and therefore are restricted to small pot studies, mainly with young plants. Conversely, rhizotron techniques lack the spatial resolution of imaging but can be used in field studies, and allow for repeated observations of root activity at the interface of the viewing window with the soil media at a constant location [1]. However, the presence of an artificial interface can lead to abnormal root activity at the interface. Additionally, as mini-rhizotron tubes are often installed in the field at angles of up to 45° to the ground surface, they can encourage vertical root tracking [3]. While some studies have investigated the bias created by the non-horizontal installation of mini-rhizotron tubes and methods to correct this bias [6], it is generally accepted that horizontally installed mini-rhizotrons are preferred for the spatial assessment of root growth over time.

In 1995, a large, field-based rhizo-lysimeter complex was constructed in an agricultural field at Charles Sturt University’s (CSU) farm at Wagga Wagga, in south-eastern Australia. The design of this facility permitted lateral access to the subsurface of each monolith, which could allow for the lateral installation of suction cups for soil solute sampling, horizontal placement of mini-rhizotron tubes for quantification of root growth [7,8] and a networked TDR for simultaneous measurement of soil water content. Since construction, several major research projects have been conducted, to investigate a typical agricultural pasture-crop rotation with the intent of understanding the implications for root growth and water use by the sequence of crops within the rotation. The objective of this manuscript, therefore, is to describe the operation of this facility, its capacity, and sensitivity in measuring plant root system development as well as quantifying the hydrologic behaviour of soils. Specifically, we consider the strengths and weaknesses of such a facility with the intention of informing the design of future rhizo-lysimeters.

## Results

### Quantitative analysis of root growth

We report on results from two major studies conducted in the rhizo-lysimeter to simulate the prevailing mixed farming rotation, using species of agronomic importance and growing in two contrasting soils. Over the period 1999–2003, lucerne was grown as a perennial pasture, followed by a series of annual, agricultural crops over the period 2004–2006; a rotation typical of the region.

### Root growth of lucerne

The study conducted over 1999–2003 focussed on the establishment pattern of lucerne roots, and their use of water in relation to depth, as affected by summer rainfall.

**Figure 1:**
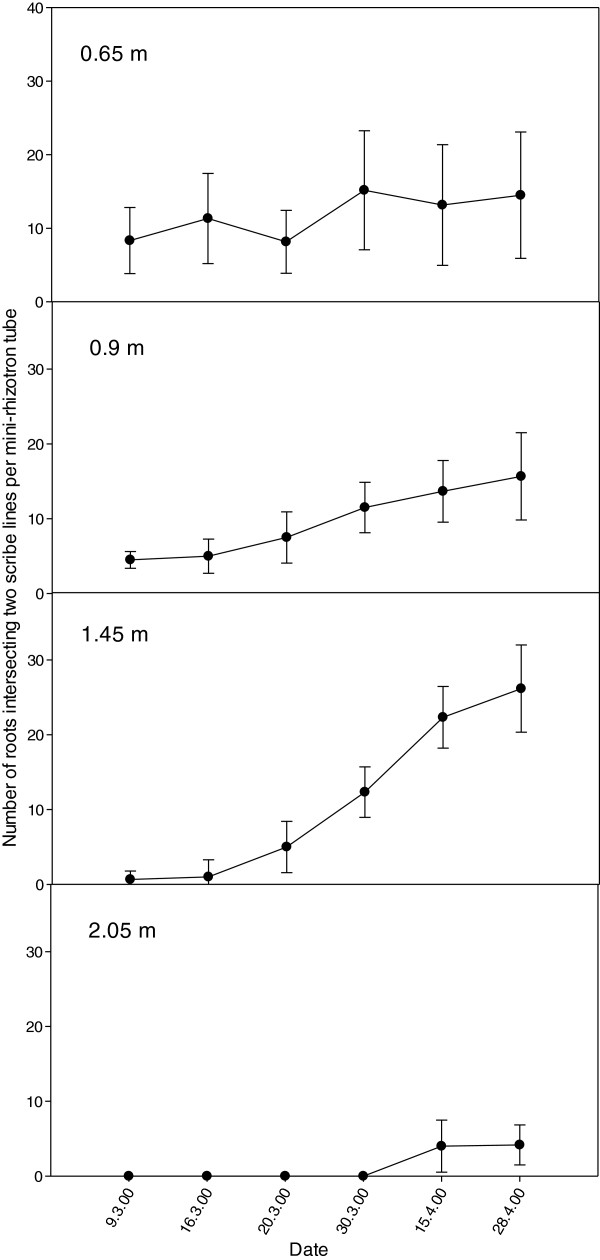
**Number of lucerne root intersecting score lines on mini-rhizotron tubes at four depths during 2000.** Each data point represents the mean of data collected from six cores and bars reflect the standard error for each mean.

**Figure 2:**
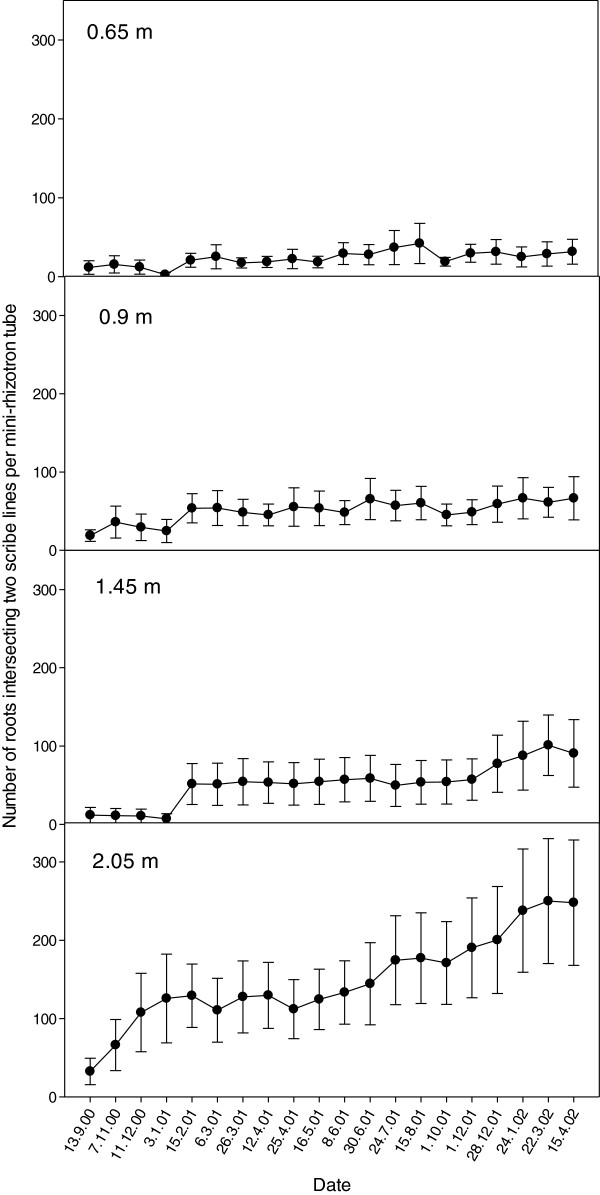
**Number of lucerne root intersecting score lines on mini-rhizotron tubes at four depths from 2000 to 2002.** Each data point represents the mean of data collected from six cores and the bars reflect the standard error for each mean. It is important to note that the lower number of points counted over the first four periods (September 9 2000 to January 1 2001) were counted using a shorter borescope and using a conversion function calculated for each tube using data collected on February 15 2001 using both the shorter and longer boroecope.

The development of the lucerne root system during season one, in relation to soil depth and surface watering is shown in Figure 1, while development over the two subsequent years (September 2000 – April 2003) is shown in Figure 2. The observations made over the duration of the study indicate that lucerne roots proliferated at shallow depths (0.65 and 0.9 m) in year 1 (Figure 1), and in this season at depths greater than 1 m, mainly in response to the application of surface water in February 2000 and April 2000. In subsequent seasons, further growth of lucerne roots were mainly deeper (2.05 m) in the soil (Figure 2).

A limited number of studies have investigated root growth of lucerne in a variety of soils in the northern hemisphere, and in general, showed that the abundance of fine roots, occurring in the surface layer (0 – 0.3 m), taper off as soil depth increases e.g. Luo et al. [9]. In contrast, observational data presented from our study indicated the reverse (Figure 2). However, as water was supplied to the deep subsoil (2.4 m), with the soil water potential maintained between −10 and −18 kPa in each core at a depth of 2.05 m, the prolific root growth observed at this depth in the later years of this study was attributed to their unrestricted access to deep subsoil water at this time and to the lower soil strength associated with moist soil. These results differ from those previously reported under typical field conditions when no access to a water table existed [9], but illustrate the degree of control and quantification of root growth offered by facilities such as these in contrast to field-based studies.

### Error analysis

**Figure 3:**
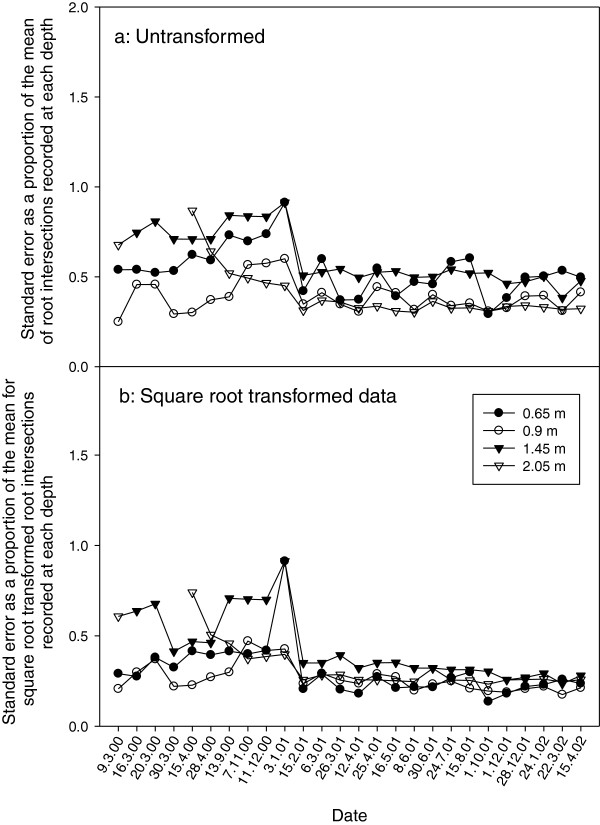
**Analysis of the standard error as a proportion of the mean for lucerne root intersection data from 2000–2002. (a)** Standard error as a proportion of the mean for untransformed data. **(b)** Standard error as a proportion of the mean for square-root transformed data.

While the use of mini-rhizotrons allowed for repeated root observations, a proportion of error about the mean of the six replicate cores could be attributed to this technique for assessment of root growth. Error bars about each point in Figures 1 and 2 increased in magnitude with increasing mean root number and also with increasing depth. However, analysis of the magnitude of standard error about the mean, as a proportion of the mean (SE/M) (Figure 3a), indicated that this statistic was relatively stable, particularly in 2001 & 2002. Closer examination of Figure 3a showed that for the roots in the shallower soil layer (0.65 m & 0.9 m), for the duration of the experiment, SE/M was relatively constant, and that the increase in SE/M in both layers at the sampling dates from September 13, 2000 in year 1 until 3 January 3, 2001 in year 2 corresponded with a period when a short (35 cm) borescope was used to assess root intersections, while the regular borescope was under repair (Figure 3a). At this time, root counts collected using the 0.3 m borescope were converted to root counts across the entire observation tube (0.76 m), by means of a regression coefficient obtained using data captured on the Feburary 15 2001 (22 months after sowing) from all tubes using both borescopes as:

$$y=-6.13+42.7\times R^{2}=0.31$$

where y = estimated number of root intersections across the whole core and × is the number of root intersections observed using the short borescope. The low R^2^ in this relationship indicates that the shorter borescope was a poor alternative for the longer instrument, confirming the heterogeneity of root growth in soil at these depths as a product of the roots tendency to congregate in zones of fractured soil or biochannels [10]. Our findings indicated that the evidence of root activity in a sole location is not a reliable predictor of root activity elsewhere in the same horizontal plane.

Unlike the magnitude of the error seen in shallower layers, the SE/M was comparatively high in the first season in the deeper layers (1.45 & 2.05 m), which is likely indicative of a sparse root system early in the establishment phase of a perennial plant but later, like in the upper layers, SE became a smaller proportion of the mean. The reduction in variability in later seasons was probably due to plant modification of the soil environment over time, such as cracking of soil in response to plant water uptake and soil drying, as well as the creation of biopores after the senescence of roots [11,12]. While the magnitude of error is a primary consideration, transforming the raw data, using a square root function, reduced the ratio of standard error to the mean substantially (Figure 3b). While the apparent standard error for each mean appeared to increase with depth, comparing square root transformed SE/M, showed that regardless of depth, standard error as a proportion of the mean was similar (Figure 3b), particularly in later seasons (e.g. years 2 and 3 (2001 and 2002). For consistent estimates of root growth, the expression of SE/M over all depths indicated that six replicates of each treatment was likely adequate, a finding which was comparable with findings of O’Toole and Bland [13]. The residual error reflected, in part, the relatively low volume of soil sampled at any particular depth with mini-rhizotrons (about 6%), and of the tendency of roots to clump in zones of reduced physical resistance [11,12], particularly in deeper soil layers. Consequently, further reductions in error using this technique are probably more difficult to obtain. Although larger volumes of soil would be useful to sample for more precise root growth assessment, the time consuming nature and cost of assessment in using sampling procedures other than mini-rhizotrons to assess growth would seriously limit most researcher’s capacity to perform this work. Therefore, the use of mini-rhizotrons for root growth assessment appears a reasonable compromise to obtain reliable root data.

### Root growth of annual crops following lucerne

The subsequent study, over the period 2004–2006 investigated root development and water-use behaviour of crops succeeding lucerne in the rotation, and used some soil cores from the previous study.

**Figure 4:**
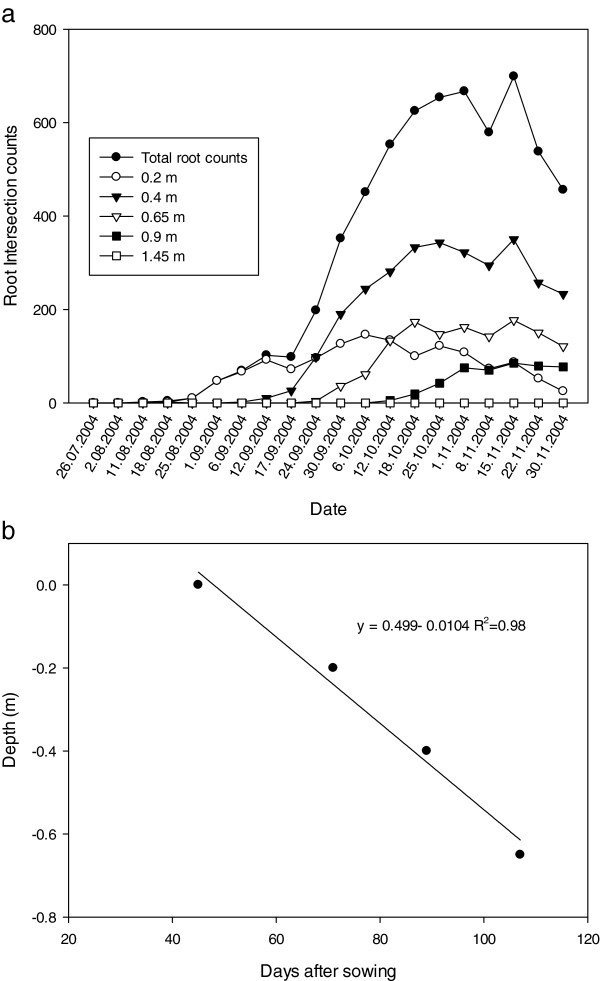
**Canola root activity in 2004 using mini-rhizotrons at six depths in the red Kandasol. (a)** The total number and number of canola root intersections counted at each depths as a function of time after sowing. **(b)** The time taken after sowing for canola roots to achieve the critical depths of 0.2, 0.4, 0.65 and 0.9 m. In the equation in figure b; y is the depth of roots (m) and x is the day of observation after sowing.

**Figure 5:**
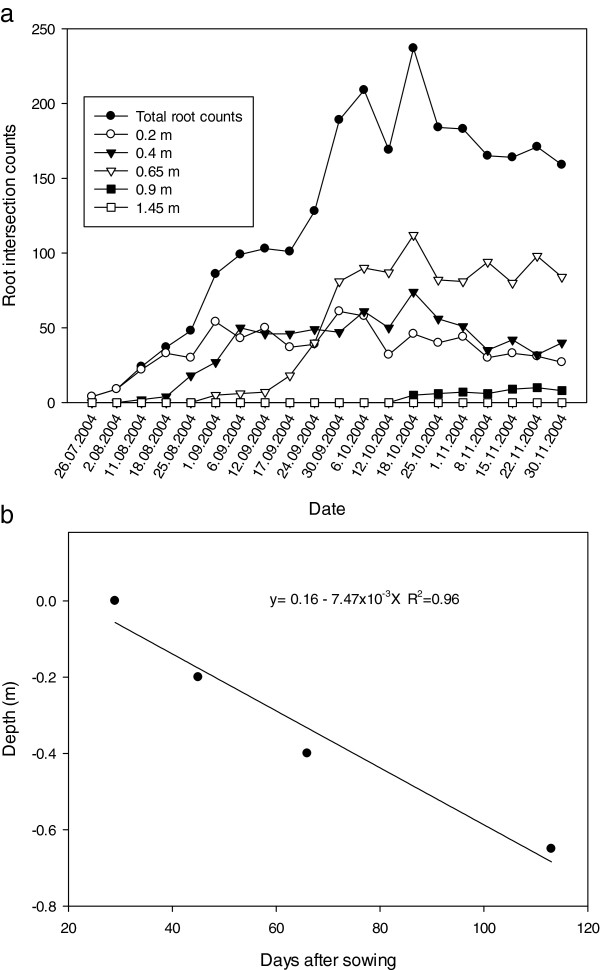
**Wheat root activity in 2004 using mini-rhizotrons at six depths in the red Kandasol. (a)** The total number and number of wheat root intersections counted at each depths as a function of time after sowing. **(b)** The time taken after sowing for wheat roots to achieve the critical depths of 0.2, 0.4, 0.65 and 0.9 m. In the equation in figure b; y is the depth of roots (m) and x is the day of observation after sowing.

Over the crop growth season, root development was monitored several times per week at depths of 0.2, 0.4, 0.65, 0.9 1.45 and 2.05 m, and expressed as: 1) total root counts per layer, 2) total root counts per core, 3) time taken (after sowing) for the first root to appear at a particular depth and, 4) the deepest observed root growth. In a comparison of the two annual crops which are commonly grown in rotation with lucerne, canola produced a more extensive root system than wheat (approximately 700 root intersections per core for canola compared with 235 root intersections for wheat), but wheat produced maximal root number several weeks earlier than canola (October 18 2004 wheat; November 1 2004 canola) (Figures 4a &5a). Further examination indicated differential patterns of root growth existed between the two crops. In relation to soil depth, canola produced a greater proportion of roots in the 0.4 m layer; in contrast, wheat favoured the development of roots deeper in the soil profile (0.65 m) (Figure 4a &5a). Maximum root numbers developed for each crop at each depth were achieved about the same time after sowing (e.g. canola and wheat; 100 & 94 DAS at 0.2 m, 120 & 113 DAS at 0.4 m; 113 & 113 DAS at 0.65 m; 140 & 148 DAS at 0.9 m respectively), with no root growth observed at soil depths below 90 cm. This indicated the distribution of roots occurred above 1 m in both annual crops during the course of this particular growing season.

Linear regression analysis indicated that for each crop, the relationship between root elongation rate and depth was linear (Figure 4b &5b), and that the rate of elongation of canola roots (1.04 × 10^–2^ m day^–1^) was similar to the rate of elongation of wheat roots (7.47 × 10^–3^ m day^–1^), with both achieving a maximum observed root depth at approximately the same time (DAS 107 canola; DAS 113 wheat). These measured rates of elongation compare well with estimates for other winter growing crops such as barley and chickpea, which ranged from 1.40 to 2.43 × 10^–2^ m day^–1^ and 1.60 to 2.36 × 10^–2^ m day^–1^ respectively, when measured using the water extraction front velocity [14], a less direct method of measuring downward progress of roots. But, these values were considerably less than values recorded for summer growing crops such as sunflower, soybean, maize and peanut, all of which had water extraction front velocities of 4.4 × 10^–2^; 3.4 × 10^–2^; 3.0 × 10^–2^; and 2.3 × 10^–2^ m day^–1^ respectively [15].

### TDR and soil hydrology

**Figure 6:**
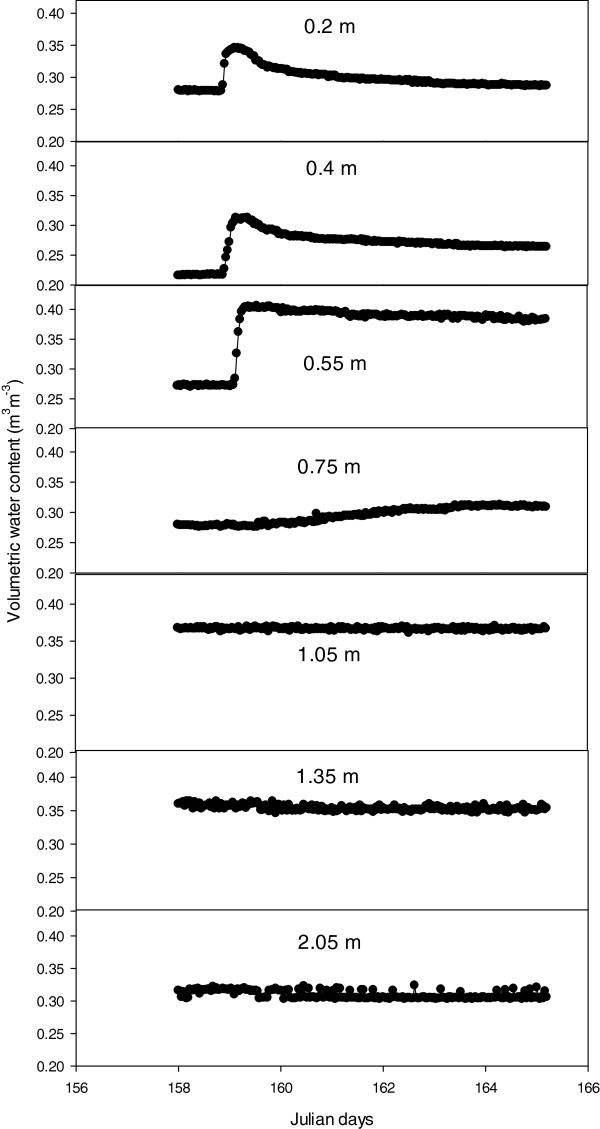
The change in volumetric soil water content of a red Kandasol at seven depths after 49.7 mm of simulated rainfall.

In a study to calibrate the TDR and to assess water infiltration in relation to depth, water added on the 2 November 2007 infiltrated the soil to a depth of 1.20 m, with little change at depths of 1.5 and 2 m (Figure 6). The average change in soil water content in each core increased by an average of 51.2 mm with standard error of 2.2 mm, from the application of 49.6 mm of simulated rainfall. Despite reference in the literature to a diversion of the dielectric constant (Ka) in some soils from the Topp’s curve [16,17], the TDR system was able to capture and document the relative change in soil water content, and illustrated its suitability for quantifying plant water uptake from soil layer, as well as drainage and water redistribution between soil layers in these soils.

Based on standard errors, the TDR system was able to predict a change in water content over the entire core, with a precision of within 5% of the amount of water applied to each soil core. This error could be further reduced by increasing the number of TDR probes per core, so that the depth increment between probes was reduced and precision improved. The improvement in the level of precision offered under this condition might allow for estimations of evapotranspiration (Et) to be made at a level of precision, approaching that offered by weighing lysimeter facilities.

## Discussion

Rhizo-lysimetric facilities are an ideal facility to undertake research of edaphic influences on plant root performance, as the level of experimental precision available exceeds that normally available from field studies. Additionally, having access to undisturbed cores with horizontally mounted observation tubes and with TDR capability, temporal root development *in situ* and resource use can be quantified at predetermined depths without the risk of root tracking occurring. This allows agricultural systems to be studied over a number of years as occurs in mixed-farming rotations, where the legacy of a crop in one season can be followed and attributed directly to the development and growth of succeeding crops.

### Methodology considerations

#### Design considerations

The Charles Sturt University facility offers several design advantages. The fully incorporated structural design used mass-produced concrete products for use as silos, and concrete in-filled reinforced keys between each silo to support the roof without the need for pillars, both of which are attributed to the low construction costs associated with building the present facility. Additionally, this design ensured good contact between the rear of each silo and the surrounding soil which enabled the soil temperatures in each core to be maintained near that of the field soils which surround the facility.

However, in spite of the temperature regulation advantage, the current rhizo-lysimeter design reduced access to each individual core as only 13% of each core was accessible from the underground chamber. This may not be an impediment for the location of certain instruments which are entirely buried, but for equipment requiring horizontal access via the underground chamber, limited vertical access could result in a vertical plane of weakness due to the vertical placement of equipment, and increase the risk of occurrence of anomalous root behaviour. Alternately, an open plan facility where there is 360° access to each core, such as in the facility at Lincoln University in Christchurch, New Zealand, would improve access and enable more uniform placement of instrumentation to lessen vertical bias, but may make the soil cores more vulnerable to temperature within the underground chamber and be less representative of the surrounding soil.

#### Assessment of root growth using mini-rhizotrons

**Figure 7:**
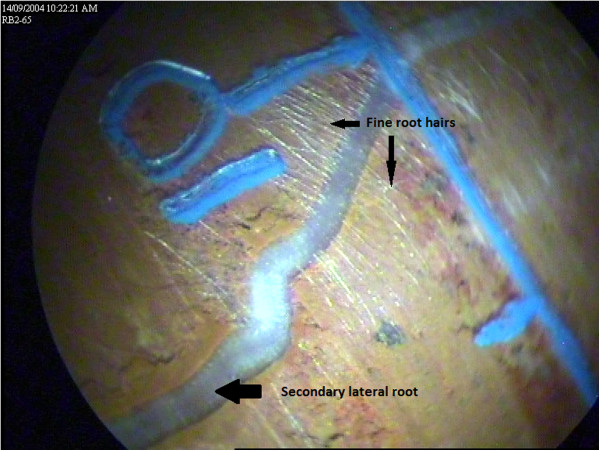
**Photograph of a wheat root at a depth of 0.65 m crossing a mini-rhizotron scribe line in a red Kandasol.** Arrows indicate a secondary lateral root and root hairs. The indentations on the scribe line indicate a distance of 10 mm. Photograph taken 14 September 2004.

**Figure 8:**
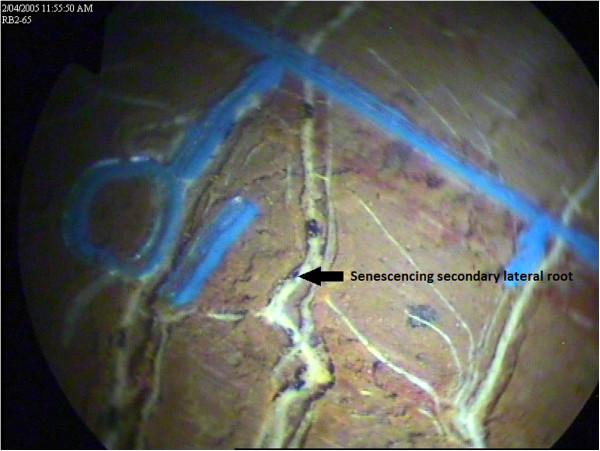
**Photograph of a senescing wheat root taken in the same location as figure 7.** Arrows indicate a senescing secondary lateral root. The indentations on the scribe line indicate a distance of 10 mm. Photograph taken 20 April 2005.

Mini-rhizotrons are a simple method to quantify *in situ,* temporal root development and senescence at pre-determined depths (see Figures 7 &8). However, as this technique makes spatial inferences about root systems from discrete observations, it is important that soil about the mini-rhizotrons are uncompromised, so that root activity in this zone represents probable root activity in the bulk soil. As with any soil research, in using mini-rhizotrons there is a risk of modifying the soil environment as may occur either during installation, or as a result of their presence of equipment which may affect subsequent root activity within the vicinity of the mini-rhizotron. Profligate root activity in the vicinity of root observation tubes has previously been reported but, mainly involves vertical or off-vertical mounted tubes where roots may be induced to track downward [3]. Carefully installed, horizontally-mounted tubes are unlikely to experience this effect. But more serious however, is the risk of soil disturbance either via fracturing or compaction that may occur in soil surrounding the mini-rhizotron during their installation. While damage may occur, careful installation into moist soil where the moisture content approaches field capacity and using installation procedures and tools such as in Box et al. [4] which cuts and removes excavated soil into the coring tube, tends to minimize this risk. Consequently from our experience in the present facility, we have not seen any evidence of imperfect installation influencing subsequent root activity, and believe the installation technique adopted was sufficient. Additionally, whereas vertically and off-vertically mounted observation tubes can only observe roots in a defined spatial location, horizontally installed tubes offer an addition spatial dimension as observations are made along a transect at a fixed depth. From our calculations, we estimate the field of view from each mini-rhizotron tubes to be about 6% of the soil volume at each depth. As a consequence, these observations have allowed for spatial root behaviour such as root clumping around soil cracks or pre-existing root channels to be quantified.

Several methods have been proposed to quantify observations of root activity using rhizotrons or mini-rhizotrons: (1) enumeration of intersections of roots with horizontal lines on the wall of the observations vessel; (2) enumeration of intersections of roots with a grid pattern scribed to the surface of the rhizotron, and; (3) enumeration of the number of root members intercepted at the wall of the rhizotron [18]. Using the CSU rhizo-lysimeter, Hoffmann [19] observed lucerne (*Medicago sativa)* root numbers by root intersection of scribed reference lines on each mini-rhizotron tube over the three year study, and inferred root development at the four depths studied. This approach was adopted since it has been reported that the number of roots intersecting a reference point on a rhizotron wall is less influenced by conditions at the wall surface than are estimations of root intensity [3,20]. Methods to extrapolate root intersection data to more tangible root growth parameters such as root length have been proposed [21,22], and are based on the assumption of homogeneity in the soil medium. However, undisturbed soils and in particular subsoils, are not homogeneous as their physico-chemical properties can change markedly over time [13] and distance, and roots are often constrained within biopores and other zones of weakness [11,12,23]. The assumption of homogeneity will in most instances give unreliable estimates of root length with a large error term comprising both experimental and computational error. Conversely, other research has shown good linearity between mini-rhizotron root numbers and root length for a range of perennial woody species [24], but report the relationship for each species to be empirical. Yet, such reports on the existence of a linear relationship between root intersections and root activity imply that the quantification of root intersections and its analysis is perhaps a good indicator of root activity in relation to genotype.

Currently the CSU facility uses two different endoscopes for viewing roots depending on the depth of the observation tubes. In the two upper observation tubes (0.2 m and 0.4 m depth), where vertical access to the tube is required, a flexible endoscope (Olympus IF8C5-30) has been extensively use. At deeper depths, where horizontal access is enabled via the underground chamber, a rigid borescope (Olympus R080-084-090-50) is used. While the flexible endoscope is perhaps more difficult to use as it does not have rotational flexibility of the optic fibre, and requires the operator to manually rotate the entire scope in moving from one reference line to the other, optically there is little difference between the two. However, a new range of industrial videoscope are becoming commercially available which feature more flexible fibreoptics, trigger grip handles and controls, and integrated cameras, and these will greatly lessen the demand on the operator and ease the routine collection of this data.

#### Estimation of soil water

Early research in the present facility relied on the neutron moisture meter to quantify change in soil water content in each profile. The transition from neutron emission to TDR has produced numerous advantages, but several disadvantages have emerged.

**Figure 9:**
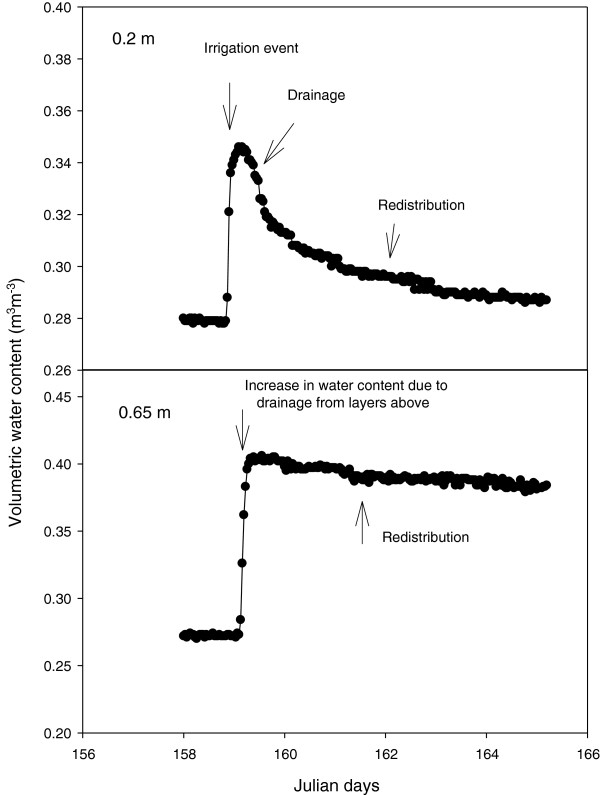
TDR trace from a single core at two depths (0.2 and 0.4 m) after a major irrigation event where evaporation was prevented from occurring.

TDR is commonly used to quantify soil water content [25,26]. While neutron emission is an excellent technique for quantifying soil water content, TDR technology offers other advantages including installation at remote field sites, networking through a series of tiered multiplexers [27,28], and a high resolution capacity, coupled with the high frequency of measurement which allows for infiltration, drainage events and redistribution events to be observed (e.g. Figure 9). However, salt concentration and soil temperature can limit the applicability of TDR to measure Ka of soil as in saline soils; the waveform tends not to be reflected due to salt, short-circuiting the sensor [29], while temperature can affect the waveform [30]. Additionally soils with high clay content confound Topp’s equation for the determination of soil water content [16,17].

To overcome the effect of high salt contents, several authors suggest sheathing the sensor to insulate the waveguide [28,31]. Mojid et al. [32] showed polyethylene to satisfactorily insulate the wire, allowing the endpoint to the signal to be resolved. In the CSU facility, increasing concentrations of salts in the red Kandasol [33] and at all depth in the grey Vertosol [33] necessitated coating the middle wire with electrical heat-shrink insulation. This treatment resulted in reasonable reflectance of the signals from the coated electrode so suitable estimates of Ka could be determined.

However, both soil types in the CSU facility were dominated by the presence of substantial amounts of clay. This high concentration of clay prevented our use of the universal calibration to measure absolute soil volumetric water content. *In situ* calibration of TDR revealed that error estimates of actual soil water content at various depths in both soil types was high. Roth et al. [16] and Bridge et al. [17] reported in high clay content soils that Ka as estimated by TDR, frequently deviated from the Topp curve. Bridge et al. [17] indicated that this was due to the unique dielectric differences between *bound water* (mono-layer adsorbed) @ Ka =3.2 compared with *free water* at 20°C @ Ka=80.4. As clay soils have a high surface area in contrast to sandy or loam soils [17], they contain vastly greater amounts of bound water compared with sandy textured soils. While bound (or adhesion) water is reflected uniformly in the calculation of soil water content, its lower Ka biases the integrated estimate of Ka for the soil layer, but once all solid surfaces have an adhering monolayer of water, further increases in soil water occur as free (unbound) water, which normally results in a linear increase in Ka of the soil until saturation occurs, and allows change in soil water content to be estimated accurately [17]. Consistent with this hypothesis, TDR in the CSU facility, while unable to predict absolute volumetric water content of each of the layers, was able to measure with great precision changes in free (or unbound) water, which is water which normally enters the soil through rainfall or acquired by plants as transpiration and is hence of agricultural significance.

#### Robustness of the system design

Many lysimeter facilities tend to use large soil monoliths which have a large evaporative surface. For example, Schneider and Howell [34] reviewed 10 different facilities for measuring E_t_ of intact ecosystems, with surface areas ranging from 2.25 – 12.6 m^2^. A preference for larger monoliths exists as their ability to mimic field conditions increases as their edge to area ratio declines and hence the boundary influence decreases. This is in stark contrast to the small pot sizes (e.g., 0.015 m^2^) that are commonly used in controlled environment studies, often with disturbed soils, and whose relationship to field conditions is questionable [35]. In contrast, the comparatively larger size of the undisturbed soil cores used in the present facility (0.45 m^2^), represents a reasonable compromise between sizes sufficiently large to reflect accurately represent field conditions, while still being of a size that doesn’t impede collection from the field at a reasonable cost. Larger undisturbed soil cores would be extremely difficult to collect intact from the field from an engineering perspective. Further, the precision of TDR, as was demonstrated in this study, can provide useful estimates of core E_t_ based on calculations of soil water deficits at multiple depths. Our multi-year experimental results demonstrate clearly that this system can provide robust measures of root growth, water use and E_t_, under conditions representative of the field and in relation to the succession of crops.

## Conclusion

The Charles Sturt University lysimeter in Wagga Wagga NSW Australia has proven to be a versatile experimental facility, which contains monoliths of soils collected from two locations remote from the facility. The lysimeter is unique due to its capability to contribute to our understanding of below-ground, temporal ecology of agriculturally-modified environments. Our particular interest in this field of research has in the past been motivated by the increasing prevalence and severity of dryland salinization in highly modified catchments in southern Australia. In addition, an understanding of the hydrologic impact associated with the conversion of a catchment from perennial, native vegetation to short-lived, annual crops and pastures is of particular relevance. In this context, the lysimeter has been an invaluable tool to study the root-zone and rhizosphere of crops or pastures over time and to assess the legacy of that historical sequence on current crop behaviour. Statistical analyses of our observations on root behaviour indicate that the history of preceding species may have an enduring influence on the root behaviour of subsequent species. Further, the legacy of an individual species may subsequently affect the soil architecture affecting the hydrological behaviour of the regolith. These long-term studies will be the subject of subsequent publications.

Despite research aimed at understanding the hydrological footprint of agricultural systems, a decade or more of drought in southern Australia has caused a change in our research focus, with climate change and plant adaptation to water deficit stress becoming of increasing interest. This facility will assume an increasingly important role in the examination of the kinetics of root behaviour, with questions related to the depth of rooting, the chemical nature of organic matter as deposited by roots, patterns of root turnover, and relationships with soil microflora and root decay rates emerging as issues of immediate relevance. Pursuit of these interests may lead to a philosophical change in the management of the facility, however, to date, the cores have been preserved in time, and physical sampling of soils has been discouraged. With the emergence of a need for more information on the carbon sequestration potential of agricultural systems, and a more thorough understanding of rhizosphere processes, there will be a great demand to sample the soils within each core and for methods to enable this to occur, while preserving the crop rotation legacy.

## Methods

### Location and description of the research facility

The facility is located in a 15 ha agricultural paddock on the Charles Sturt University Farm at Wagga Wagga, New South Wales, Australia (Latitude 35° 03’ 15.40” S Longitude 147° 20’ 14.53” E**)** at an elevation of 219 m above sea level. The site is serviced by single phase 240 V AC power supply, and underground high pressure water mains, telephone and a high speed data communications cable.

**Figure 10:**
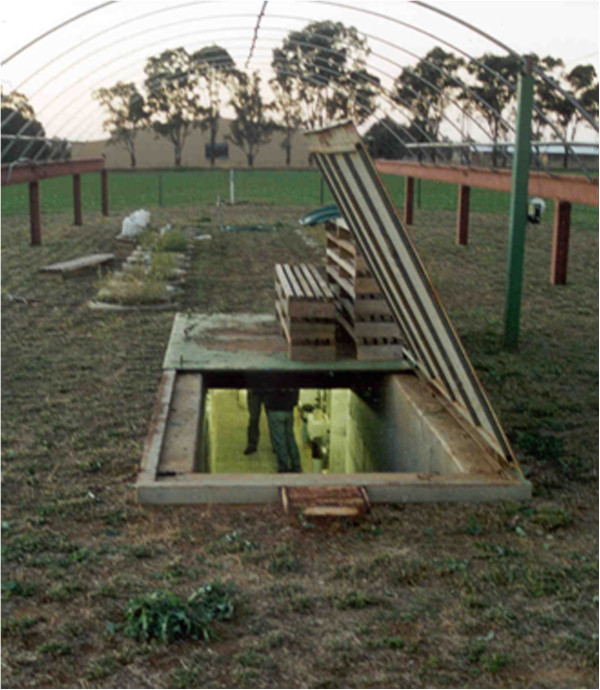
**View of the surface and underground work area of the CSU rhizolysimeter complex.** In the background, silos containing cores are growing lucerne, while in the foreground, the entrance and stairwell which provide access to the underground laboratory are visible.

**Figure 11:**
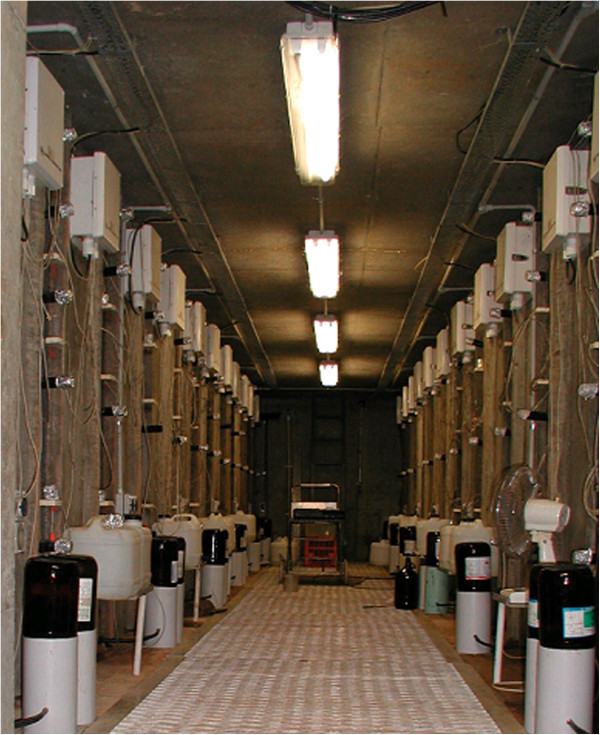
**The underground laboratory beneath the rhizo-lysimeter.** The white boxes at the top of each core contain multiplexers and network the TDR signals emanating from each core. The black tubes covered with aluminium caps protruding from each core are exposed section of the mini-rhizotron root observation tubes.

**Figure 12:**
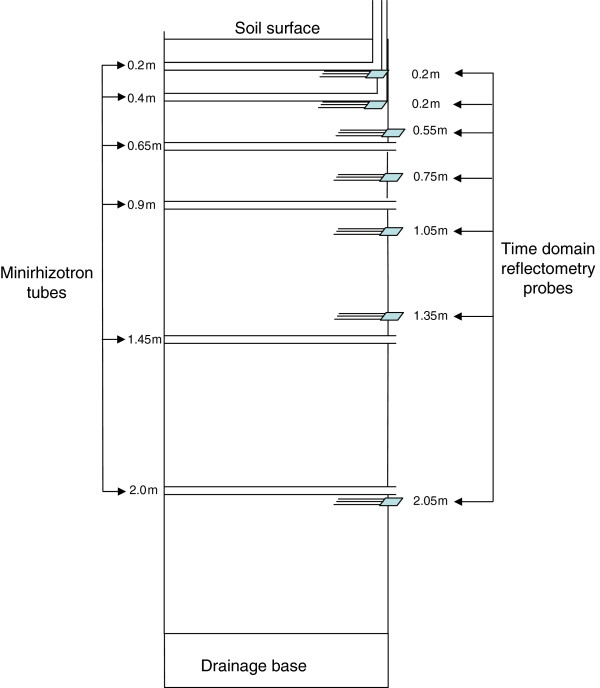
**Cross-section view of each soil core illustrating the placement of instrumentation.** TDR probes were located at approximately 0.2, 0.4, 0.55, 0.75, 1.05, 1.45, 2.0 m beneath the soil surface. Mini-rhizotron placements were at approximately 0.2, 0.4, 0.65, 0.9, 1.45 and 2.05 m beneath the soil surface. The two mini-rhizotron tubes closest to the soil surface were installed in 2003 and were accessible by a vertical riser using a flexible endoscope.

**Figure 13:**
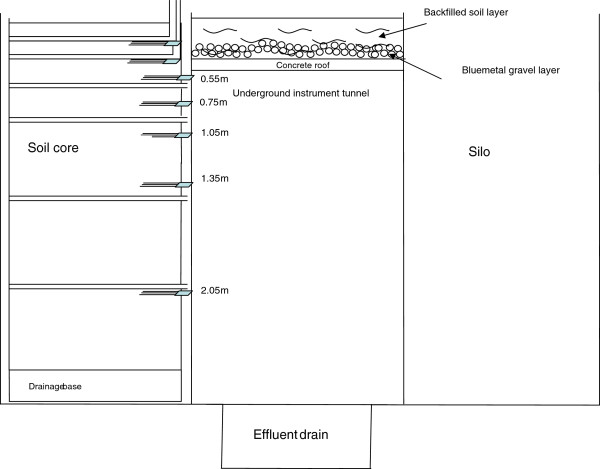
Cross-section view of the rhizo-lysimeter tunnel illustrating the nature of the roof and soil above the roof and the fit of a core in a silo.

The research facility comprises two rows of 12 concrete silos separated by a 1.2 m underground corridor (Figures 10 &11). The facility was constructed after excavation, using pre-fabricated upturned concrete culverts and silos, modified concrete pipes to house the cylinder-containing soil core, and a poured concrete stairwell, instrument alcove, end walls and roof. An overhead gantry crane which runs on parallel tracks on either side of, and for 10 m beyond the facility, enables soil cores collected from the field and transported in via truck to be lowered into their silos or moved from one silo to another. Each silo is 2.5 m deep × 0.8 m diameter and has an opening from the underground chamber at a depth of 0.5 m which allows access to the side of the core directly for observations and measurements. As the roof of the facility is 0.5 m thick, including a 0.25 m layer of friable soil, it precluded lateral access to the surface 0.5 m of each core. In later experiments, to enable root observations to be made in this zone, horizontally-mounted mini-rhizotron tubes with a riser to the soil surface were installed before the cores were lowered into their silos (see Figure 12 &13).

A rainout shelter which was installed using the rail system of the gantry crane to provide protection to the soil cores. The rainout shelter was constructed from hoops of RHS (0.05 m × 0.02 m) galvanized steel rolled into semi-circular hoops which were then covered with a polyethylene film, similar to that used in hoop houses. While there was some interception of photosynthetically-active radiation by the film, the amount transmitted and arriving at the plant canopy was still in excess of plant needs (between 60 and 85% of incoming radiation).

### Instrumentation

#### Mini-rhizotrons and optics

Visualization of vertical root development, clumping behaviour and root turnover was enabled using a series of hollow, horizontal, clear polycarbonate mini-rhizotron tubes (0.75 m (L) × 0.042 m (dia) id × 0.003 m (wall thickness)) which were installed into pre-cored holes. Prior to insertion, two scribe lines were etched along the axis of each tube at positions of 10 and 2 o’clock (300° and 60° respectively), indented every cm, and dyed blue to allow for colour contrast against a soil background. For research undertaken during the period of 1999–2003, four mini-rhizotron tubes were installed per core at depths of 0.65, 0.90, 1.45 and 2.0 m beneath the soil surface, and in subsequent years, two additional mini-rhizotron tubes were installed at depths of 0.2 and 0.4 m. These tubes were installed at soil depths where horizontal access was not possible due to the height of the roof, but access was allowed by attaching a 90^0^ angle PVC tube to their end and connecting vertical PVC risers which rose above the soil surface (Figure 12).

At all depths, the procedure of Box et al. [4] was employed to install mini-rhizotrons. At each depth a pilot hole was drilled using a rotary hammer drill, and this hole was enlarged using a sampling tube fitted with a cutting tip similar to that described by Box et al. [4], and designed so that soil displaced by the cutting tip was compressed toward the centre of the sampling tube which minimized the compression of soil external to the tube. Following the removal of the coring tube, the internal surface of the hole was gently abraded using a fine wire brush to remove any smeared or soil reoriented by the sample tube and ensured good contact between the mini-rhizotron and the surrounding soil.

Roots at depths of 0.2 and 0.4 m beneath the soil surface were viewed using a 3 m flexible fiberscope (IF8C5-30; Olympus Australia Pty Ltd) with an optical adapter (AT60S/NF; Olympus Australia Pty Ltd) and a side-viewing window with a field of view of 60° was used. At other depths, a 0.84 m (8 mm diameter) rigid borescope (R080-084-090-50; Olympus Australia Pty Ltd) with a lateral view of 90° and a 50° field view was used. Roots in cores were illuminated by attaching a Microlight 150 light source (Fibreoptics Lightguides, Australia) fitted with a 150 watt halogen lamp to the scope. A digital camera (WAT-202D; Watec Co., LtD., Japan) was attached to the rigid borescope and connected to a TV monitor (TFTV535BK Palsonic Australia) on which the root images were displayed. The camera was also connected to a laptop computer by way of a video capture card (VCE-PRP; ImperX Incorporated; Boca Raton, USA), and digital images or film clips could be captured and analysed at a later date.

At all depths, mini-rhizotron tubes were visually scanned for the number of roots intersecting the two etched lines at a given time, based on a theory developed by Lang and Melhuish [21]; in later experiments with annual crop species, the time taken to reach particular depths was also recorded.

#### Soil water determination: neutron moisture meter

In all cores, a single aluminium access tubes (1.8 m deep × 0.05 m diameter), positioned slightly off-centre to avoid interaction with mini-rhizotron were installed into hand augured holes and grouted into place with a kaolin slurry using the procedure of Greacen [36]. The neutron moisture meter (503 DR Hydroprobe, CPN International) was calibrated for each soil type by the installation of similar access tubes at an undisturbed site adjacent to where the cores were originally collected, at both the wet and dry ends of the range of soil water content [19]. The time for each count of the Neutron Moisture Meter was 16 seconds.

#### Soil water determination: time domain reflectometry

In 2003, seven 0.3 m three wire type TDR probes (0.0048 m diameter and 0.045 m spacing) (CS605, Campbell Scientific Pty Ltd) were installed laterally into each core at depths of 0.2, 0.4, 0.55, 0.75, 1.05, 1.35 and 2.0 m and connected to a centralized TDR network (Figure 11). The TDR system included a Tektronics 1502C Metallic Cable Tester (Tektronics, Inc) with an effective bandwidth of 1 GHz equipped with a communications interface (SDM1502; Campbell Scientific Pty Ltd). The system was controlled with a CR10X datalogger (Campbell Scientific Pty Ltd) and a series of twenty-six integrated multiplexers (SDMX50; Campbell Scientific Pty Ltd) which provided a network for measuring soil water content in each core at each depth.

TDR probes were inserted horizontally into 28 cm pre-drilled, slightly undersized pilot holes, as Rothe et al. [37] found that pushing probes into undisturbed soil increased the bulk density of soil immediately surrounding the probe and resulted in a lower estimation of soil water content. At the 20 and 40 cm depths, the TDR probe heads were entirely buried in soil, (note detail in Figure 12) with only the cable protruding, while at greater depths, probes were inserted into soil such that the wires on each probe were more than 0.03 m away from the outer wall to avoid interference from the steel cylinder so that the probe head partially protruded outside of the steel casing.

The networked system had the capacity to analyse soil water content in each core at each depth at a maximum rate of once every 48 minutes using the Topps calibration equation [26], soil volumetric water content was determined at each depth.

### Collection of undisturbed cores from the field

Soil monoliths were collected from the field in rolled steel cylinders (2.4 m × 0.76 m dia) made from 6 mm mild, galvanized steel plate. The cutting end of each core was reinforced by welding on an additional 6 mm mild steel rim cut at a 45° chamfer.

**Figure 14:**
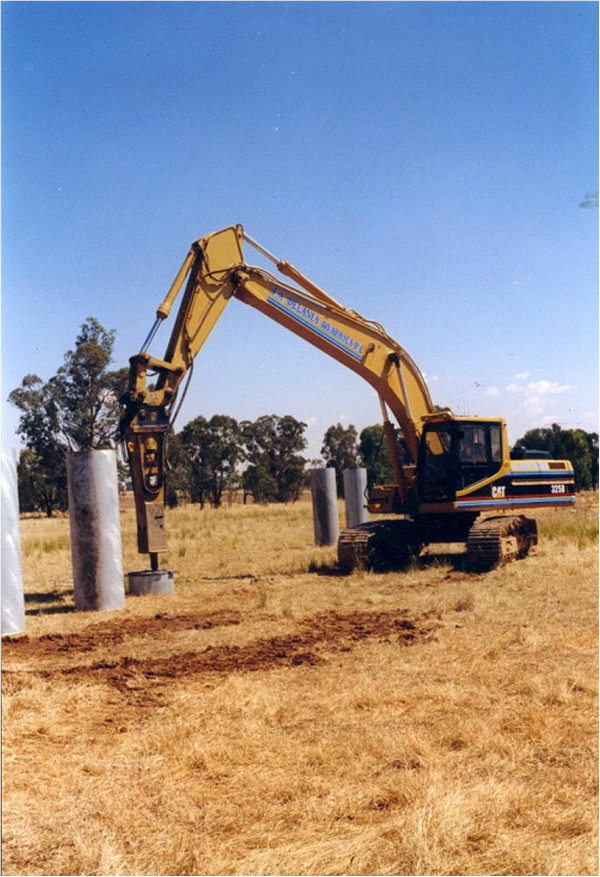
Excavator hammering the steel core in to collect the undisturbed soil core.

At the time of collection, soil cores were collected from the field intact. Soil cores were collected by using a mild steel pressing plate 0.8 m in diameter and 0.05 m thick with a machine steel centre, on top of the core and using the boom, the weight of the excavator was brought to bear on the core. Once the core had been pushed vertically into the ground to a depth of about 1 – 1.5 m (Figure 14), the perimeter soil around the matrix of cores was excavated to a depth of 2.5 m and the core hammered in to a depth of 2.35 m using a hydraulic hammer attached to the boom of the excavator. The cylinder and soil core were removed using the boom of the excavator and lifting chains, and once removed a sand-filled drainage base welded to the core. The sand-filled steel drainage base contained a perforated plastic tube which protruded from the side of the base and enabled drainage water to be collected.

### Root growth of lucerne (1999–2003)

This study was conducted using two soils; a red Kandasol [33], a medium clay, composed principally of illite and kaolin clay particles, and physically and chemically fertile; a grey Vertosol [33] a heavy smectite-dominated, sodic clay, known to physically and chemically impede the production of agriculturally-important pasture and crop species. Pre-inoculated lucerne (*Medicago sativa)* seed, either Pioneer L34 or Pioneer L90, were sown into hand-cultivated, pre-fertilized cores in 17 cm-spaced rows at a rate equivalent to 4 kg ha^–1^ in May 1999. After establishment, plants were thinned to about 120 seedlings m^–2^ and over the ensuing study period, further self-thinning occurred such that by year three, lucerne populations were approximately 45 plants m^–2^.

Over the subsequent three years, root development and turnover at depths of 0.65, 0.9, 1.45 or 2.05 m beneath the soil surface were monitored, and the change in soil water content in the upper 1.8 m monitored using a neutron moisture meter. During the first season (summer of 1999–2000), surface water was applied using a drip irrigation system to the lucerne-containing cores on two occasions: February 20 2000, where 120 mm of water was applied, and on April 4 2000, where 50 mm of water was applied. In subsequent years, lucerne cores were watered during the winter and spring (May-October) by rainfall (379.6 mm and 248.2 mm during 2000 and 2001 respectively). In contrast, during the summer-autumn period of 2000–01 and 2001–02, cores were surface watered using drip irrigation at the rate of 25 mm of water per core per week. Additionally, during the period from February 2001 – May 2002, the deep subsoils of all cores (2.4 m) were continuously watered via capillary rise from a permanent and unlimited supply of water into the drainage bases.

### Root growth of annual crops following lucerne (2004–2006)

Treatments selected in year one (2004) were either canola (*Brassica napus* cv Rivette) or wheat (*Triticum aestivum* cv Diamond Bird) following lucerne or in similar cores with no prior history of lucerne. The wheat and canola cultivars selected tolerated a short growing season and were, in the case of canola, resistant to blackleg. In year two, cores which in the previous year had been sown to canola were sown with wheat, or visa versa.

For the duration of the experiment, each core received an allocation of water per month equivalent to the long-term average rainfall calculated for the Wagga Wagga region for that particular month. Monthly water applications were split into two even allocations per core per month and applied as during the lucerne phase via a drip irrigation system.

Over the subsequent two years, root development at depths of 0.2, 0.4, 0.65, 0.9, 1.45 or 2.05 m beneath the soil surface were monitored using a flexible endoscope (0.2 and 0.4 m depths only) and a rigid borescope (0.65, 0.9, 1.45 and 2.05 m), on a weekly basis and the change in soil water content in the upper 2 m monitored using TDR.

### TDR and soil hydrology

Six plant-free cores containing the red Kandasol were calibrated on several occasions by adding 22.5 L of water to each core, the equivalent of 49.6 mm of rainfall, using four 2 L hr^–1^ drippers and by eliminating soil evaporation. The change in volumetric soil water content over the entire soil monolith, the volumetric soil water content (Θ_v_) 24 hours prior to and after the addition of water was estimated at each depth, and from this, the change in Θ_v_ at each depth calculated. In scaling up to the monolith, the change in Θ_v_ at each probe was assumed to be representative of the soil water content up to half way between this and the next probe, and hence, an average change in Θ_v_ for a particular depth horizon calculated. The average of each horizon was then summed and the difference in core Θ_v_ prior to and 24 hours after watering calculated. The results of the calibration conducted over October and November 2008 for six cores are given in Table 1.

**Table 1:** Change in volumetric soil water content (mm) in six red Kandasol monoliths as estimated for each soil layer using a 0.3 m unbalanced, mid-mounted TDR in response to a simulated rainfall event of 49.6 mm

**Horizon depth (m)**	**Core number**						
	1	2	3	4	5	6	Mean
	mm	mm	mm	mm	mm	mm	mm
0 – 0.3	15	11.5	10.2	11.7	16.2	11.1	12.6
0.3 – 0.5	5.6	6.2	5.6	5.4	4.4	5.6	5.5
0.5 – 0.75	6	8	7.8	6.	8.3	8.8	7.5
0.75 – 1.15	8.4	22	13.6	16.4	15.2	16.4	15.3
1.15 – 1.35	2.8	7.4	10	5.6	13	6.2	7.5
1.35 – 1.75	0.4	1.5	1.6	0.4	-1.6	0.4	-0.6
1.75 – 2.25	9	4.5	1	1	2	3	3.4
Total	47.2	58.1	46.7	46.5	57.5	51.5	51.3

## Abbreviations

TDR: Time domain reflectometry;SE/M: Standard error about the mean as a proportion of the mean;DAS: Days after sowing;Ka: Dielectric constant;RHS: Rolled hollow section;Θv: Soil volumetric water content;Et: Evapotranspiration

## Competing interests

The authors declare they have no competing interests.

## Authors’ contributions

PLE obtained the grant and built the facility and conceived with LJW the concept of the manuscript and drafted the manuscript. PLE conceived the idea and received the grant, and JH ran the lucerne study for which he was awarded a PhD. PLE conceived the idea and received the grant and SM ran the canola and wheat study. All authors approved and commented on the manuscript. PLE and LAW revised the manuscript.
